# Pathogens Causing Pediatric Community Acquired Urinary Tract Infections and Their Increasing Antimicrobial Resistance: A Nationwide Study

**DOI:** 10.3390/pathogens13030201

**Published:** 2024-02-24

**Authors:** Vered Shkalim Zemer, Shai Ashkenazi, Yoel Levinsky, Yael Richenberg, Eyal Jacobson, Shay Nathanson, Tzippy Shochat, Shiri Kushnir, Moriya Cohen, Avner Herman Cohen

**Affiliations:** 1 Clalit Health Services, Petach Tikva 4900000, Israel; 2 Faculty of Medicine, Tel Aviv University, Tel Aviv 6997801, Israel; yoel.lvn@gmail.com (Y.L.); hermanc@post.tau.ac.il (A.H.C.); 3 Adelson School of Medicine, Ariel University, Ariel 4070000, Israel; shaias@ariel.ac.il; 4 Pediatric Rheumatology Unit, Schneider Children’s Medical Center of Israel, Petach Tikva 4920235, Israel; 5 Department of Pediatrics B, Schneider Children’s Medical Center of Israel, Petach Tikva 49420235, Israel; 6 Dan-Petach Tikva District, Clalit Health Services, Petach Tikva 4972339, Israel; dpyael@clalit.org.il (Y.R.); eyaljac@clalit.org.il (E.J.); shayna@clalit.org.il (S.N.); 7 Statistical Consultation Unit, Rabin Medical Center, Beilinson Hospital, Petach Tikva 4941492, Israel; tzippysh@clalit.org.il; 8 Research Center, Rabin Medical Center, Beilinson Hospital, Petach Tikva 4941492, Israel; shiriku1@clalit.org.il; 9 Microbiology Unit, Ariel University, Ariel 4070000, Israel; moriya1cohen@gmail.com; 10 Pediatric Ambulatory Community Clinic, Petach Tikva 4931807, Israel

**Keywords:** urinary tract infections, children, uropathogens, *Estherichia coli*, antibiotic treatment, antibiotic resistance, guidelines

## Abstract

Urinary tract infections (UTIs) in childhood are common and are associated with considerable acute morbidity and long-term complications. The need for updated data to optimize empiric antibiotic therapy is crucial. We aimed to investigate the pathogens causing pediatric community acquired UTIs, their correlation with demographic characteristics, and trends in their antimicrobial resistance. This nationwide cross-sectional study included all 53,203 children (<18 years) diagnosed with UTI in community outpatient clinics in the following selected years: 2007, 2011, 2015, 2019 and 2021. *Escherichia coli* (*E. coli*) (82.1%) was the most common uropathogen, followed by *Enterobacter*, *Klebsiella*, *Proteus*, *Pseudomonas*, and *Enterococcus* species. The bacterial distribution displayed statistically significant (*p* < 0.0001) gender- and sector-specific patterns with a higher relative prevalence of non-*E. coli* UTI in Jewish and males. The rate of extended-spectrum beta-lactamase-positive *E. coli* increased substantially and significantly (*p* < 0.001) from only 6.1% in 2007 to 25.4% in 2021. Most non-*E. coli* uropathogens exhibited resistance to commonly used empiric antibiotics for UTIs in children. These findings are significant in guiding optimal empiric antibiotic treatment for pediatric community acquired UTIs. The resistance of uropathogens to antimicrobials is region- and time-dependent. Therefore, the periodic and local assessment of antibiotic resistance trends is essential to update guidelines and provide the most appropriate antibacterial therapy for children with UTIs.

## 1. Introduction

Urinary tract infection (UTI) is one of the most common bacterial infections during childhood [1,2,3]. The prevalence of UTIs varies according to age, gender, and geographical location. Up to 8% of children experience at least one UTI between the ages of 1 month and 11 years, and up to 30% of infants and children experience recurrent infections during the first 6 to 12 months after an initial UTI [4,5]. Pediatric UTIs are associated with significant acute morbidity and may cause long-term complications such as arterial hypertension, renal scarring, and even chronic renal failure [1,6].

Most pediatric UTIs are caused by Gram-negative coliform bacteria arising from fecal flora that colonize the perineum and ascend the urinary tract. *Escherichia coli* (*E. coli*) is the most common organism causing pediatric UTI. Other common uropathogens include *Klebsiella*, *Proteus*, *Enterobacter*, and *Enterococcus species* (spp.) [7].

In pediatric patients with suspected UTI, antibiotic treatment is usually started empirically, namely before urine culture results are available. The inappropriate and frequent use of antibiotics has long-term effects on the human microbiome and induces bacterial resistance [8]. Appropriate treatment of UTIs has become challenging due to the high resistance of uropathogens to commonly prescribed antimicrobial agents [9] and the globally increasing prevalence of multidrug-resistant organisms causing UTIs [10]. Therefore, continuously updated examination of bacteria causing UTIs in the various pediatric populations and their antibiotic-resistant patterns are crucial in enabling pediatricians and family physicians to choose the most appropriate empiric antibiotic treatment.

Using demographic and laboratory data over 15 years, our study aimed to explore the demographic characteristics of children with community acquired UTI, investigate the causative pathogens in this population, determine the antimicrobial resistance patterns of these pathogens, and elucidate the trends during the study period.

## 2. Materials and Methods

### 2.1. Study Population

We conducted a nationwide retrospective cohort study based on data from Clalit Health Services (CHS), which is the largest insurer-provider health maintenance organization in Israel. It insures about 5 million (54%) of the Israeli population. CHS is geographically a nationwide health maintenance organization covering more than half of the Israeli population and thus represents all sectors of the population. CHS comprises a comprehensive computerized database, which is continuously updated regarding patients’ sociodemographics, outpatient medical visits, laboratory results, hospitalizations, diagnoses, and medications prescribed and purchased.

This study included the entire Israeli pediatric population (<18 years) in CHS, including neonates. All positive urine cultures processed in the microbiology laboratory of CHS that were taken from pediatric patients, who were seen in outpatient clinics for suspected UTI in five selected years, including 2007, 2011, 2015, 2019, and 2021, were included in the study. The years were selected randomly to detect changes over time in the UTI-causing bacteria, as well as in the patterns of their antibiotic resistance.

Data on children with UTI were retrieved from the electronic medical files using the CHS Data Sharing Platform powered by MDClone, https://www.mdclone.com (accessed on 15 January 2024). Parameters of age, gender, and date of urine culture were recorded. Israel’s population accounts for 9.842 million people, with diverse ethnic and cultural subpopulations. Approximately 74% of the population are Jews, 21% are Arabs, and 5% are of other ethnicities (Israel Central Bureau for Statistics, available at: https://www.cbs.gov.il/he/pages/default.aspx, accessed on 10 February 2024). We, therefore, divided our study population into two distinct demographic groups, which we referred to as “sectors”: Jews and Arabs. Results of urine culture and antibiotic susceptibility of the isolated pathogens were recorded.

The study was approved by the local institutional review board for human studies (approval number 0096-16-COM2).

### 2.2. Laboratory Analysis

All positive urine cultures in the five selected years, 2007, 2011, 2015, 2019, and 2021, were examined at the microbiology laboratory of Clalit Central Laboratory, Israel. We excluded from the study the following urine culture results: 1. negative cultures; 2. growth of lower than the standard quantity of 50,000 colony-forming units (CFUs)/mL; 3. growth of bacteria that are considered contaminants; and 4. growth of more than one bacterium. Samples taken less than 7 days apart were considered as a single episode. After standard cleaning, urine samples were obtained from patients as recommended by the guidelines of the American Academy of Pediatrics [11] by bladder catheterization or midstream urine. The diagnosis of UTI was made according to the recommended laboratory criteria [11]. These included pyuria (positive leukocyte esterase or nitrite on dipstick or >5 WBC/high power field on centrifuged urine microscopy) and/or bacteriuria and ≥50,000 CFUs/mL growth of a single uropathogen [11]. The disc diffusion method was used on isolated pathogens to perform antimicrobial susceptibility testing by means of a panel of antimicrobial substances, as recommended [12]. The interpretation of susceptibility tests was performed according to the recommended criteria [12]. Antimicrobial susceptibility testing was performed for ampicillin, amoxicillin/clavulanate, cephalexin, cefuroxime, ceftriaxone, nitrofurantoin, trimethoprim/sulfamethoxazole (TMP/SMX), ciprofloxacin, gentamicin, and amikacin. Uropathogens that were intermediate (I) or resistant (R) to an antibiotic agent were considered resistant.

Categorization of *E*. *coli* as extended-spectrum β-lactamase (ESBL)-producers was performed as described [13,14]. Namely, ESBL production was determined in two stages. Initially, isolates were screened using an automated identification system (Vitek 2); then ESBL-positive isolates were confirmed using disc diffusion testing. To detect ESBLs, discs of ceftazidime and ceftriaxone were placed 30 mm from an amoxicillin/clavulanate (20/10 mg) disc. A >5 mm increase in zone diameter for either antimicrobial agent tested in combination with clavulanic acid versus its zone when tested alone confirmed an ESBL-producing organism [13,14].

### 2.3. Statistical Analysis

Statistical analysis of the study results was performed using SAS Software, Version 9.4. For descriptive statistics, continuous variables are presented as mean ± standard deviation or median and IQR; categorical variables are presented as number (%). Fisher’s exact test was used to compare the values of categorical variables among the study groups. Two-sided *p*-values < 0.05 were considered statistically significant.

## 3. Results

### 3.1. Demographic Characteristics

The study population included a total of 53,203 children with UTIs. The demographic characteristics of the children with positive urine cultures diagnosed in 2007, 2011, 2015, 2019, and 2021 are presented in Table 1. Significant gender-based differences were observed, especially in the older age group. Females were more commonly presented than males: 49,274 (92.6%) vs. 3929 (7.4%). Among children younger than 3 years, 85.7% were females and 14.3% were males, while among those aged 3 to 18 years, 95.5% were females and 4.5% were males. The mean age of females was 7.2 ± 5.4 years, and that of males was 5.6 ± 6.3 years (*p* < 0.0001). Regarding the sector, 69.2% of the patients were Jewish and 30.8% were Arabs.

### 3.2. Isolated Urinary Pathogens

Gram-negative bacteria constituted the largest group with a prevalence of 96.8%. *E. coli* was the most common bacterial uropathogen (82.1%) and was isolated in all age groups. Less frequent Gram-negative bacteria were *Proteus* (7.1%), *Pseudomonas* (3.7%), *Citrobacter* (1.5%), *Enterobacter* (1.5%), and *Klebsiella* spp. (0.9%). Only 3.2% of isolates were Enterococcus spp., a Gram-positive microorganism.

**Figure 1:**
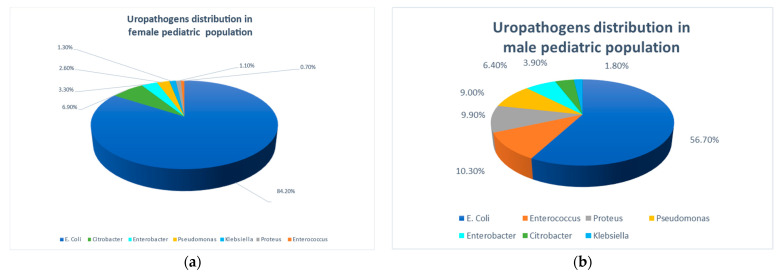
Pathogens causing community acquired urinary tract infections in the study population. (**a**) In females; (**b**) In males.

Statistically significant gender-specific differences were noted in the relative prevalence of the various uropathogens (*p* < 0.0001), as demonstrated in Figure 1. *E. coli* was more prevalent among females compared to males (84.2% and 56.7%, respectively). Conversely, the other less common non-*E. coli* uropathogens were more common in males than in females: *Proteus* spp. 9.9% vs. 6.9%; *Pseudomonas* spp. 9% vs. 3.3%; and *Enterococcus* spp. 10.3% vs. 2.6%. *Enterobacter*, *Citrobacter,* and *Klebsiella* spp. were also relatively more prevalent among males than females.

**Figure 2:**
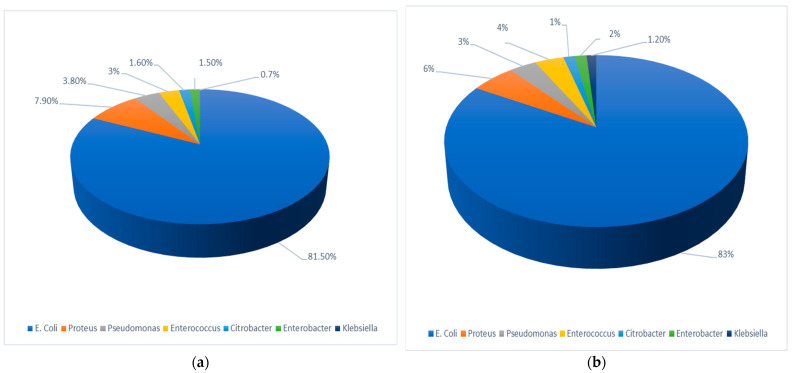
Pathogens causing community acquired urinary tract infection in the study population. (**a**) In the Jewish pediatric population; (**b**) In the Arab pediatric population.

There were also statistically significant sector-specific differences in the relative prevalence of the various uropathogens, namely between Jewish and Arab children (*p* < 0.0001) as demonstrated in Figure 2. *E. coli* was the most prevalent uropathogen in both sectors, but its relative prevalence was lower in children in the Jewish sector than in the Arab sector (81.5% vs. 83.4%). *Proteus* spp. were the second most prevalent in both Jewish (7.9%) and Arab (5.5%) children; *Pseudomonas* spp. were the third most prevalent (3.8%), and *Enterococcus* spp. (3%) were the fourth most common uropathogens in Jewish children, while *Enterococcus* spp. were the third most prevalent (3.6%) and *Pseudomonas* spp. were the fourth most prevalent uropathogens (3.5%) in Arab children.

### 3.3. Antimicrobial Resistance Profiles of Bacterial Uropathogens

**Figure 3:**
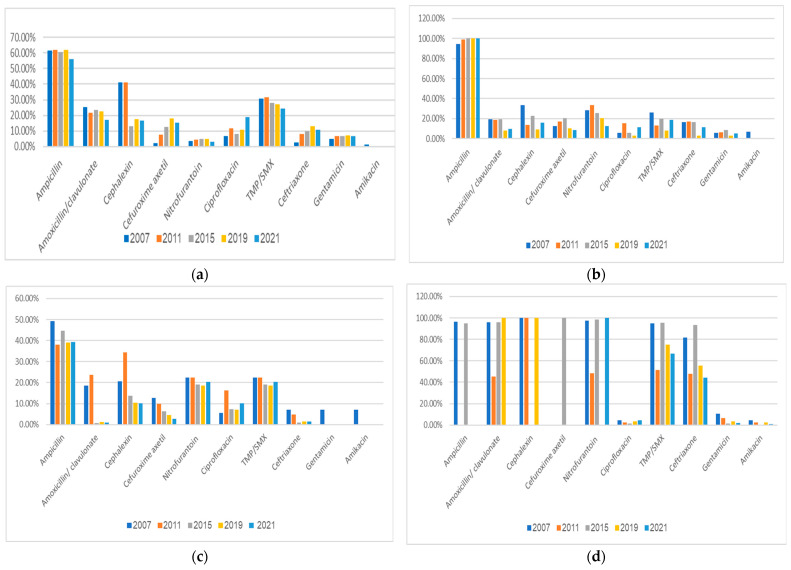
Rates of antimicrobial resistance of uropathogens to various antimicrobial agents during the study period. (**a**) *E. coli*; (**b**) *Klebsiella* spp.; (**c**) *Proteus* spp.; (**d**) *Pseudomonas* spp. TMP/SMX, trimethoprim/sulfamethoxazole.

The antimicrobial resistance profiles of the main UTI-causing bacteria during the study period are shown in Figure 3. *E. coli* isolates had a very high resistance rate of >55% to ampicillin and a high resistance rate of ≥25% to TMP/SMX throughout the study period (Figure 3a). Of note is the significantly increased resistance of *E. coli* isolates to ciprofloxacin (from 6.9% in 2007 to 19% in 2021), to cefuroxime (from 2.4% in 2007 to 15.4% in 2021), and to ceftriaxone (2.6% in 2007 and 10.9% in 2021). There was a mildly increased resistance to gentamicin (5.1% in 2007 and 6.9% in 2021), with most strains being susceptible to amikacin (~1% resistance) and nitrofurantoin (~3% resistance). The resistance of *E. coli* decreased over the study period to amoxicillin/clavulanate. Additional details are shown in Figure 3a.

The antimicrobial resistance profile of *Klebsiella* spp. over the study period is shown in Figure 3b. Of note is the near complete resistance of *Klebsiella* isolates to ampicillin (94.3% in 2007, 100% in 2015 to 2021). About 20% were resistant to TMP/SMX. *Klebsiella* spp. isolates were most susceptible to the aminoglycosides gentamicin and amikacin. Additional details are shown in Figure 3b.

The antimicrobial resistance profile of *Proteus* spp. over the study period is shown in Figure 3c. Of note is the highest resistance rate of *Proteus* spp. isolates to ampicillin (~40%) and a high resistance rate of ~20% to TMP/SMX and nitrofurantoin. Most *Proteus* isolates were susceptible to amikacin, gentamicin, and ceftriaxone.

The antimicrobial resistance profile of *Pseudomonas* spp. over the study period is shown in Figure 3d. As expected, most of the *Pseudomonas* isolates were resistant to ampicillin, amoxicillin/clavulanate, nitrofurantoin, TMP/SMX, cephalexin, cefuroxime, and ceftriaxone. *Pseudomonas* isolates were mostly susceptible to ciprofloxacin, amikacin, and gentamicin.

### 3.4. Frequency of ESBL-Producing E. coli

**Figure 4:**
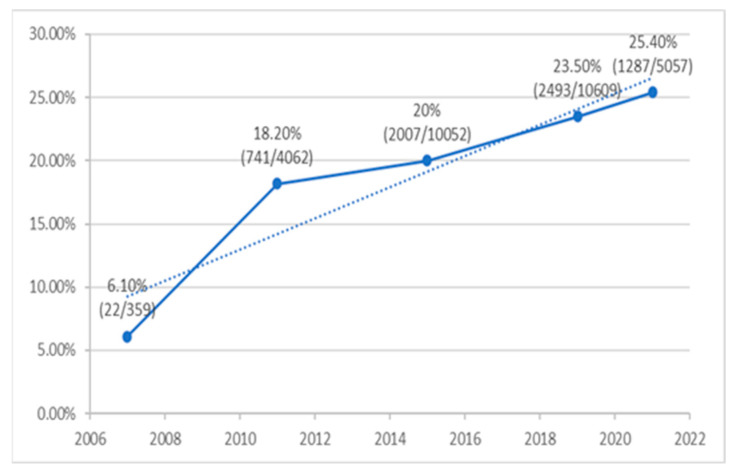
Percentage (number positive/number tested) of ESB-producing *E. coli* causing community acquired pediatric UTI infection during the study period.

We investigated the prevalence of ESBL-production by *E. coli*, the most common uropathogen, with high numbers of isolates each year throughout the study period; the results are presented in Figure 4. It is evident that the rate of ESBL-positive *E. coli* of all the UTI-isolated *E. coli* increased steadily and considerably over the study years, from only 6.1% in 2007 to 20% in 2015; while in 2021, the rates peaked at 25.4% (r 0.934, *p* < 0.001).

## 4. Discussion

The present population-based nationwide study highlights significant findings regarding the pathogens causing pediatric community acquired UTIs and their increasing antimicrobial resistance over 15 years. These findings include:

1. Gender and sector differences in uropathogens: a significantly higher relative prevalence of E. *coli* in females and the Arab population.
2. A significantly increased resistance of *E. coli* isolates during the 15-year study period to ciprofloxacin, cefuroxime, ceftriaxone, and gentamicin, with most non-*E. coli* uropathogens resistant to the antimicrobial agents often used empirically to treat UTIs in children.
3. The rate of ESBL-positive *E. coli* causing UTI increased considerably and significantly over the study years, from only 6.1% in 2007 to 25.4% in 2021.

These findings are significant because antimicrobial agents for suspected UTI are usually given empirically before culture results are available. Indeed, the empiric choice of the most appropriate antibiotic agent for UTI requires updated knowledge of the causes of the infection and the status of antimicrobial resistance among uropathogens. The findings are also crucial for developing guidelines for the empiric antibiotic treatment of UTI [1,3,11]. However, as data on the causative uropathogens and their antimicrobial susceptibility patterns vary by location and time, updated local data are of major importance [3,10,15].

*E. coli* was the most common bacterial pathogen causing community acquired UTI in our population, which is consistent with previous studies in multiple locations [15,16,17,18,19,20,21,22,23,24,25]. Graif et al. investigated bacterial uropathogens among 1056 children aged <15 years in Northern Israel between 2010 and 2017. They found that *E. coli* was the leading pathogen (detected in 76% of urine cultures), followed by *Klebsiella* (7%), *Enterococcus* (5.8%), and *Proteus* (4%) spp. [15]. Eramenko et al. [16] examined the bacterial distribution among children aged 3 months to 18 years diagnosed with UTI and treated as outpatients in a large community clinic in Israel between 2015 and 2017. *E. coli* was the most common causative bacteria (78.1%), followed by *Proteus* spp. (11.2%), *Klebsiella pneumoniae* (3.9%), and *Enterococcus* spp. (3.4%) [16]. Similarly, in our study, *E. coli* was the most common bacterial uropathogen (82.1%), while *Proteus* (7.1%) and *Pseudomonas* spp. (3.7%) were the next most common UTI-causing bacteria.

Previous studies on community acquired UTIs conducted in multiple countries worldwide demonstrated that 44.1% to 94.4% of the cases were females [15,16,17,18,19,20,21,22,23,24,25]. Likewise, in our study, 92.6% of the participants aged <18 years were females, which was the predominant gender in all age groups. In our study, we identified gender-specific pathogen distribution, which was statistically significant (*p* < 0.0001). Our finding of a higher prevalence of *E. coli* UTIs among females and a higher prevalence of *Pseudomonas* UTIs among males has been reported in other studies [24,26].

Statistically significant sector-specific uropathogenic distribution in the Israeli population, in both children and adults, has been previously reported [15,27]. Graif et al. published a single hospital-based study over 7 years that compared bacterial uropathogens isolated from Arab and Jewish pediatric populations in Israel. They found that *E. coli* was more common among children of Arab origin than Jewish origin (78.7% versus 72.2%, respectively), while *Enterococcus* spp. (7.2% compared to 4.8%), *Proteus mirabilis* (5.5% compared to 2.4%), and *Pseudomonas aeruginosa* (3.5% compared to 1.8%) were more common among Jewish children [15]. Our study confirms and expands these findings using nationwide-based data over 15 years. Because of the retrospective nature of our study, we could establish associations, not causality. The sector differences are probably related to disparities in antibiotic consumption and underlying urinary malformations. Indeed, it has been previously shown by us that previous antibiotic use is associated with increased rates of non-*E. coli* UTI [27]. It is possible that antibiotics were used more commonly by the Jewish population, leading to a higher frequency of non-*E. coli* UTI. Regarding the gender differences, we have previously shown that underlying abnormalities of the urinary tract are associated with higher rates of non-*E. coli* UTI [27]. As UTI in boys is more often associated with underlying urinary malformation, this can explain the higher rates of non-*E. coli* UTI in boys than in girls. Additional multicenter studies are required to determine the implications of ethnic background on empiric antibiotic therapy in the Mediterranean area. Nevertheless, sector and gender background should be considered when choosing the empiric antibiotic treatment for children with UTIs.

Increasing antibiotic resistance is a major challenge that narrows the options of antibiotic agents for treating UTIs [18,28]. The reported resistance rates of bacterial uropathogens against ampicillin, one of the most frequently used empiric agents for UTI, are high worldwide [15,16,18,20,22,23,29,30], as was also noted in our study. Antimicrobial resistance of UTI-causing *E. coli*, by far the most common uropathogen, is increasing globally. A systematic review demonstrated that in countries of the Organization for Economic Cooperation and Development (OECD), the pooled resistance prevalence of pediatric UTI-causing *E. coli* was 53.4% for ampicillin, 23.6% for trimethoprim, 8.2% for amoxicillin/clavulanate, and 2.1% for ciprofloxacin. Resistance in non-OECD countries (including the Middle East) was significantly higher: 79.8% for ampicillin, 60.3% for amoxicillin/clavulanate, 26.8% for ciprofloxacin, and 17.0% for nitrofurantoin [9].

A major finding of our study is the significant steadily increased rates of ESBL-positive *E. coli* causing UTI. Similar trends have been reported in other locations [11,31]. This community acquired ESBL-producing *E. coli* is alarming and should be followed closely to identify risk factors for community acquired UTI caused by ESBL-producing *E. coli* and prevent its spread. Non-*E. coli* uropathogens are more often found in children with underlying urinary tract abnormalities and are often resistant to antimicrobial agents used routinely to treat UTIs in children, as we have shown in previous studies [27,32,33].

In 2016, the American Academy of Pediatrics published clinical guidelines regarding UTI treatment in the pediatric population [11]. They recommended that empiric therapy for UTIs should be based on local antimicrobial susceptibilities. The proposed oral treatment options were first- or second-generation cephalosporins, amoxicillin/clavulanate, and TMP/SMX. Parenteral-recommended antibiotic treatment comprised third-generation cephalosporins, gentamicin, or piperacillin until the identification of the bacterial urinary pathogen and its antimicrobial susceptibilities, when treatment can be adjusted accordingly [11]. Improved diagnosis of UTI will prevent overuse of antibiotics [11,34,35,36].

The Israel Medical Association published in 2022 guidelines for the treatment of pediatric patients (aged >3 months) with UTIs. The empiric antibiotics recommended were TMP/SMX, nitrofurantoin, or first-generation cephalosporins (cephalexin) for cystitis; in patients with pyelonephritis, it was recommended to consider empiric intravenous therapy with gentamicin (first choice) or ceftriaxone due to high bacterial resistance to oral antibiotics. Intramuscular antibiotics should be used when no other possibility exists. In community acquired pyelonephritis, oral empiric antibiotics are a possibility in patients aged >3 months, when the clinical presentation is not severe, without significant background diseases or signs of dehydration, and full compliance to oral therapy is guaranteed. For pyelonephritis, the preferable empiric oral antibiotics are second-generation cephalosporins (cefuroxime) or amoxicillin/clavulanate [37]. During most of our study period, first-generation cephalosporins (cephalexin) were most commonly used for lower UTI (cystitis), while IV treatment with gentamicin or ceftriaxone were usually used in children with pyelonephritis. The results of our study show ~30% resistance to TMP/SMX, and thus it seems less appropriate for empiric treatment.

## 5. Strengths and Limitations

The strengths of the current study are mainly the nationwide 15-year study of a high number of >53,000 children with UTI, which yields a high power to reach statistically significant differences in the relative prevalence of uropathogens by type of population and in antimicrobial resistance profiles of the pathogens. The main limitation is its retrospective nature with limited clinical data. We did not assess the presenting symptoms and severity of the UTIs, nor did we investigate the correlation between antibiotic resistance and clinical outcomes, which were beyond the scope of the present study. Also, we did not include information on the presence of urinary tract malformations and the use of prophylactic antibiotics, which can affect the bacteria causing UTIs and their antimicrobial susceptibility. Regarding the methods used to detect ESBL throughout the study period, only 3rd generation cephalosporins were used for synergy testing, but not 4th generation cephalosporins or a monobactam, that are needed to detect different ESBL-types.

## 6. Conclusions

The results of our study show that gender and sector are significantly associated with the relative prevalence of *E. coli* vs. non-*E*. *coli* uropathogens, the latter being more resistant to antimicrobial agents often used empirically to treat UTIs in children. These findings should be considered when deciding the optimal empirical antibiotic treatment for pediatric community acquired UTIs [38,39]. The resistance profiles of uropathogens to antimicrobials are region- and time-dependent. Therefore, it is prudent to test the trends of antibiotic-resistant uropathogens periodically and locally, to update guidelines and provide patients with the most appropriate antibacterial therapy for UTI [3,11,40,41,42].

## Figures and Tables

**Table 1:** Demographic characteristics of children with urinary tract infections diagnosed during the study period.

		Year					Total
		2007	2011	2015	2019	2021	
**No. of Patients**		9936	10,726	12,113	11,015	9413	53,203
**Age (years)**	**Mean ± SD**	7.2 ± 5.6	7.1 ± 5.5	7.0 ± 5.5	7.0 ± 5.4	7.3 ± 5.4	7.1 ± 5.5
	**Median**	5.7	5.6	5.5	5.5	5.9	5.6
**<3 years**	**N (%)**	2979 (30%)	3145 (29.3%)	3649 (30.1%)	3223 (29.3%)	2548 (27.1%)	15,544 (29.2%)
**≥3 years**	**N (%)**	6957 (70%)	7581 (70.7%)	8464 (69.9%)	7792 (70.7%)	6865 (72.9%)	37,659 (70.8%)
**Gender**		8966 (90.2%)	9871 (92%)	11,181 (92.3%)	10,375 (94.2%)	8881 (94.4%)	49,274 (92.6%)
**Female**	**N (%)**						
**Male**	**N (%)**	970 (9.8%)	855 (8%)	932 (7.7%)	640 (5.8%)	532 (5.6%)	3929 (7.4%)
**Sector**		6698 (67.4%)	6992 (65.2%)	8443 (69.7%)	7871 (71.5%)	6812 (72.4%)	36,816 (69.2%)
**Jewish**	**N (%)**						
**Arab**	**N (%)**	3238 (32.6%)	3734 (34.8%)	3670 (30.3%)	3144 (28.5%)	2601 (27.6%)	16,387 (30.8%)

## Data Availability

All supporting data are within the article.
