# Correction: Shkalim Zemer et al. Pathogens Causing Pediatric Community Acquired Urinary Tract Infections and Their Increasing Antimicrobial Resistance: A Nationwide Study. *Pathogens* 2024, *13*, 201

**DOI:** 10.3390/pathogens14020150

**Published:** 2025-02-05

**Authors:** Vered Shkalim Zemer, Shai Ashkenazi, Yoel Levinsky, Yael Richenberg, Eyal Jacobson, Shay Nathanson, Tzippy Shochat, Shiri Kushnir, Moriya Cohen, Avner Herman Cohen

**Affiliations:** 1 Clalit Health Services, Petach Tikva 4900000, Israel; 2 Faculty of Medicine, Tel Aviv University, Tel Aviv 6997801, Israel; 3 Adelson School of Medicine, Ariel University, Ariel 4070000, Israel; 4 Pediatric Rheumatology Unit, Schneider Children’s Medical Center of Israel, Petach Tikva 4920235, Israel; 5 Department of Pediatrics B, Schneider Children’s Medical Center of Israel, Petach Tikva 49420235, Israel; 6 Dan-Petach Tikva District, Clalit Health Services, Petach Tikva 4972339, Israel; 7 Statistical Consultation Unit, Rabin Medical Center, Beilinson Hospital, Petach Tikva 4941492, Israel; 8 Research Center, Rabin Medical Center, Beilinson Hospital, Petach Tikva 4941492, Israel; 9 Microbiology Unit, Ariel University, Ariel 4070000, Israel; 10 Pediatric Ambulatory Community Clinic, Petach Tikva 4931807, Israel

## Error in Figure

In the original publication [1], there was a mistake in Figure 1. The same chart was mistakenly used for Figure 1a,b in the published paper. The correct Figure 1 is as follows:

The authors state that the scientific conclusions are unaffected. This correction was approved by the Academic Editor. The original publication has also been updated.

## Figures and Tables

**Figure 1:**
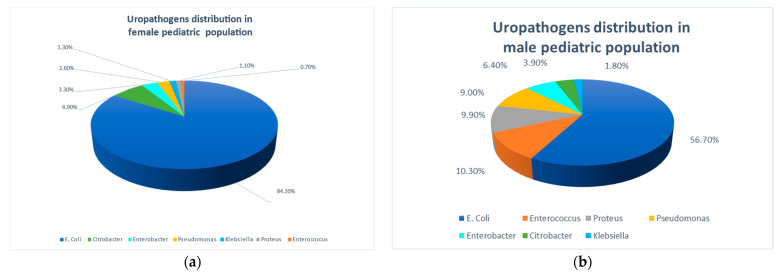
Pathogens causing community acquired urinary tract infections in the study population. (**a**) In females; (**b**) In males.
